# Science and prizes: A case for rethinking the criteria for prizes in science (and for rewarding important discoveries in bacterial physiology)

**DOI:** 10.1038/s44319-024-00091-z

**Published:** 2024-02-09

**Authors:** Vic Norris

**Affiliations:** Bacterial Communication and Anti-infection Strategies, University of Rouen, 76821 Mont Saint Aignan, France

**Keywords:** History & Philosophy of Science, Science Policy & Publishing

## Abstract

Scientific prizes award those making important discoveries, define a field, further the public understanding of science, or support young and upcoming researchers. But they should also encourage scientists to take risks and venture into the unknown.

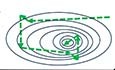

The bacterial cell cycle comprises chromosome replication, chromosome segregation and cell division. It is a major source of phenotypic diversity and an integral part of bacterial physiology. Despite its importance for a wide range of fields from ecology to human health to biotechnology, few major prizes have been awarded for studies of the bacterial cell cycle. One could make a similar case for plant science—which is enormously important for agriculture—or for ecology or evolution. Understanding this lacuna requires asking why scientific prizes exist in the first place and what problems are associated with them.

Prizes are often rooted in a particular vision of science, which sees a prize-worthy achievement as a result of impressive work and as an answer—or a new route to an answer—to a scientific question. This view has repercussions beyond just awarding a scientist for his or her work. According to Karl Popper, hypotheses that survive systematic attempts to disprove them by testing their predictions should be considered as ‘corroborated’ or ‘confirmed’, which is as close as a hypothesis can get to being true. However, many actors in politics, the media and industry often refer to scientific results and achievements to justify their activities (Klikauer, 2023) with the implicit assumption that if science says something is true it must be true—and even more so if it was awarded with a prize. This increasing trend to take science at face value—“to trust the science”—is one reason for the growing mistrust of science and scientists by the public if those achievements turn out to be preliminary or not as reliable than they first seemed to be, in particular if a prize has helped to attract public attention in the first place.

Prizes are often rooted in a particular vision of science, which sees a prize-worthy achievement as a result of impressive work…

## The function(s) of prizes

Prizes are awarded at many levels for many activities with different criteria. “The *Nobel Prize for Physiology or Medicine* is awarded for discovery of major importance in life science or medicine. Discoveries that have changed the scientific paradigm and are of great benefit for humankind are awarded the prize, whereas life-time achievements or scientific leadership cannot be considered for the *Nobel Prize*” (nobelprize.org). The purpose of the *Robert A. Welch Award in Chemistry* “is to foster and encourage basic chemical research and to recognize, in a substantial manner, the value of chemical research contributions for the benefit of humankind as set forth in the will of Robert Alonzo Welch. […] We intend that the award recognizes the contributions of an individual who has not previously been recognized in a similar manner” (welch1.org/awards/welch-award-in-chemistry). The *Helmholtz Prize* does not require prior publication of a paper; if there has been a publication, it “must have taken place within the last two years, as the *Helmholtz Prize* is awarded projects which have been completed recently” (www.helmholtz-fonds.de/helmholtz-prize/). Similarly, the *Louis Jeantet Prizes* “are not intended solely as the recognition of work that has been completed, but also to encourage the continuation of innovative research projects (www.jeantet.ch/en/louis-jeantet-prize/prix-louis-jeantet/).

To be awarded the *Fields Medal*, a recipient must be younger than 40 years on 1 January of the year in which the medal is awarded. This rule is apparently based on John Charles Fields’ wish that “while it was in recognition of work already done, it was at the same time intended to be an encouragement for further achievement on the part of the recipients and a stimulus to renewed effort on the part of others” (Riehm, 2002). The *Breakthrough Prizes in Physics, Life Sciences and Mathematics* recognize “the world’s top scientists working in the fundamental sciences”; the winners are chosen by Selection Committees composed of previous *Breakthrough Prize* laureates. They also award prizes in physics and mathematics for younger researchers who have already produced significant works, and prizes for female mathematicians who have recently completed their PhD (breakthroughprize.org). The *Turing Prize* claims to adopt a different approach: “The world faces critical challenges (such as the AI alignment problem) … We don’t have the answers …, but believe that we need new ideas, perspectives, and thinkers to solve these challenges. But they often don’t have financial support or they don’t know how to get started. That’s why we’re running the *Turing Prize*” (amturing.acm.org).

The *Lasker Awards* are intended both to highlight fundamental biological discoveries and clinical advances and to encourage public support of science (laskerfoundation.org). According to Paul Nurse, who won the Lasker Award in 1998, “Prizes are good because they get science out into the mass media and into the public” (laskerfoundation.org). This sentiment is echoed by Andreas Radbruch on the scientific advisory council of the *Robert Koch Foundation*: “The *Robert Koch* prizes make progress visible, they are a compass for future research” (www.robert-koch-stiftung.de).

## Criteria for prizes

The criteria for awarding prizes are sometimes criticised, for example, on the grounds that they reward individuals rather than groups and, in particular, those who are good at building networks (Hansson and Steinke, 2019). According to Bernard Rossier on the scientific committee of the *Louis Jeantet Foundation* “considering all the developments, the technical platforms, it becomes more and more difficult to identify individuals […] but I think there is still a lot of room for individual and important discoveries” (Breithaupt, 2005). The *Guardian*’s Robin McKie takes these criticisms further: “modern science doesn’t really have the kind of great, individual discoverers that *Nobel* laureates are generally seen as. Instead, great discoveries are products of collaboration, which means that a system that actively rewards personal achievements (and thus promotes competition) is downright dangerous” (https://www.grunge.com/172955/the-messed-up-truth-of-the-nobel-prize/).

Although one might disagree with McKie’s point that there are no longer great individual discoverers, he does have a point that promoting competitiveness comes at the expense of the collaborative spirit. While competition is healthy, too much of it favours a corrosive ‘publish or perish’ climate that puts pressure on scientists to exaggerate results or claim discoveries when there are none to be made. It encourages the construction of hierarchies or pecking orders that pervades science; it starts at graduate school and continues with the Shanghai classification of universities. In this construction of hierarchies, prizes play their part and awards are often for being good *at* science rather than being good *for* science. Such sentiments were voiced by the Russian mathematician Gregori Perelman, who refused the *Fields Medal* and a million-dollar award from the Clay Mathematics Institute: “I do not like their decision, I consider it unfair. I consider that the American mathematician Hamilton’s contribution to the solution of the problem is no less than mine” (https://mathshistory.st-andrews.ac.uk/Biographies/Perelman/).

In this construction of hierarchies, prizes play their part and awards are often for being good *at* science rather than being good *for* science.

Prizes help to define a field not just by recognising certain contributions but also by ignoring others. In other words, prizes can reinforce the *status quo* by drawing attention, funding and talent to well-established areas at the expense of others that may have an equal or even greater potential for real breakthroughs. In that regard, prizes and their selection committees run the risk of becoming victims of the *Matthew effect* and the *Shared Reality* theory. The *Matthew effect* is generally understood as “the rich get richer” while “the poor get poorer”, which translates to science as “the rarely cited become the uncited” (Caleiro, 2018). *Shared Reality Theory* states that interpersonal relationships are regulated by the degree to which people share experiences and beliefs, which, in science, describes a group of scientists who hold and share similar convictions about what is important and what is not (Conley et al, 2010).

… prizes can reinforce the *status quo* by drawing attention, funding and talent to well-established areas at the expense of others…

## Playing it safe

If the primary concern is to ‘play it safe’, to ensure that the prize really does reward a major contribution to science—which Nobel committees usually do—this often entails waiting for the endorsement by the wider community or leading experts, which can take so long that the scientist has died before the prize can be awarded. This creates the risk that important contributions to science eventually remain unrewarded, such as Rosalind Franklin’s crucial role in describing the structure of DNA, Susan Lindquist’s ground-breaking discoveries about protein folding or Rosetta Reusch’s work on the role of poly-(R)-3-hydroxybutyrate (PHB) in forming ion channels and modifying proteins. And it discourages risk-taking, paradigm-shifting science. Ludwig Fleck, a microbiologist, has explained why doing such science is difficult: “Once a structurally complete and closed system of opinions consisting of many details and relationships has been formed, it offers enduring resistance to anything that contradicts it” (Cooper, 2017). One might add that science, like nature, abhors a vacuum, which may explain why the Replicon Theory lasted unchallenged for so long (Nordstrom, 2003). One might also add that, with thousands of genes and macromolecules and post-translational modifications to manipulate, it is easy to obtain interesting and potentially prize-worthy results without challenging established knowledge or generating novel hypotheses that eventually open up new directions for research.

**Figure 1:**
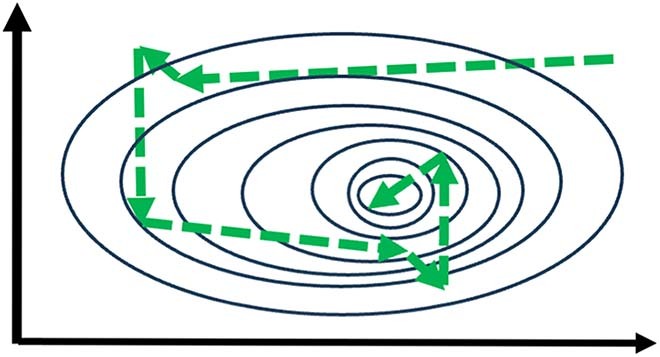
Chemotaxis. The contours describe the increasing concentration of a chemo-attractant in the middle of the plot and the dotted green arrows describe the direction of a moving bacterium. Changes in direction occur when the direction is not—or is no longer—taking the bacterium up the gradient. Science progresses in a similar way by getting it right and by getting it wrong.

Another problem with ‘playing safe’ when awarding prizes is that it is based on a misunderstanding of how science actually progresses. The risk of focussing on Newton’s “If I have seen further, it is by standing on the shoulders of Giants”, or on Kuhn’s ‘paradigm shifts’ is to overlook the dependence of scientific progress on getting it *wrong*. One might liken the progress of science to the progress of a bacterium swimming up a gradient of a chemoattractant (Fig. 1). Its short-term memory detects that the concentration of the attractant is decreasing, which increases the probability that it stops swimming in a straight line (‘run’) but instead ‘tumbles’ to allow it to swim in a new, semi-randomly chosen, direction. Put differently, in this strategy of run and tumble—which is highly effective—getting it ‘wrong’ is integral to progress. Finally, in the case of the *Breakthrough Prizes*, the alleged ‘Oscars of Science’, there is an association with celebrities and billionaires, whose values may sit uneasily with scientists.

## Discussion

Prizes can be used to reward individuals or, as in the case of the *Turing Prize*, to encourage research in a particular field, or, as in the case of the *Lasker Awards*, to increase public support for science. It is therefore surprising that no major prizes have been awarded specifically for contributions to understanding the bacterial cell cycle. It is tempting to imagine that prizes for microbial physiology would reflect the relevance of the discovery to clinical medicine and award scientists whose achievements “are of great benefit for humankind”. But while the bacterial cell cycle has crucial implications for infectious disease, human health and biotechnology, this may be too indirect for clinicians to appreciate.

Ideally, prizes like the *Lasker Awards* might help to give the general public—and perhaps some in the medical community—a better idea of how science works. They might also have the flavour of the *Turing Prize* by reflecting, anticipating and encouraging changes in a particular field. For example, our understanding of the bacterial cell cycle has changed in several major ways over recent years. The focus has shifted from the population to the single cell and from the average cell to the diversity of individual cells; the interpretative framework has shifted from a simple, unstructured bacterial cell to a *hypercomplex*, highly structured cell; the variety of techniques employed have broadened from those in molecular genetics and biochemistry to include those in physics and physical chemistry. It also raises novel questions. Why do cells regulate the cell cycles to have, for example, particular sizes and DNA/mass ratios? Why do cells bother with a cell cycle at all? What is a cell? Are there answers to these questions that would also help to answer different fundamental questions?

Ideally, prizes like the *Lasker Awards* might help to give the general public—and perhaps some in the medical community—a better idea of how science works.

## A new prize

To remedy the paucity of prizes for work related to the bacterial cell cycle, the *Charles E. Helmstetter Prize for Groundbreaking Research in Bacterial Cell Cycle Physiology* was inaugurated recently at the EMBO Workshop *Bacterial cell biophysics: DNA replication, growth, division, size and shape*, which was held in Ein Gedi, Israel (Norris and Zaritsky, 2023). It was named in honour of the person widely acknowledged as having provided the theoretical and experimental bases for our current understanding of the bacterial cell cycle. The *Charles E. Helmstetter Prize’s* ambition should be to adopt criteria that, like life itself, are diverse, dynamic and adaptive; achieving this goal may benefit from a variety of different selection committees as well as from the absence of a constraining ‘mission statement’.

The *Charles E. Helmstetter Prize’s* ambition should be to adopt criteria that, like life itself, are diverse, dynamic and adaptive…

The criteria that achievements should be long-lasting and universally accepted should be factors for awarding the *Charles E. Helmstetter Prize*. It should, however, also recognize that contributions—whether new hypotheses or exciting experimental findings—are rarely immutable or independent of their environments. Any theory that has been ‘killed’ by counterfactual results can be ‘resurrected’ by new evidence or become cannibalised by another, newer, hypothesis. For example, the disproven hypothesis that the helical growth of peptidoglycan controls the cell cycle by (Mendelson, 1982) has been resuscitated by new evidence for the shape-determining role of helical, actin-like protein filaments in *Bacillus subtilis* (Jones et al, 2001).

**Figure 2:**
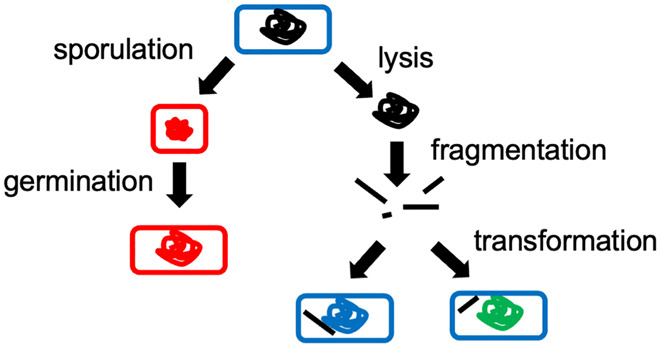
Bacterial population. In a population of bacteria, some cells sporulate (small red cell with compact red DNA) and grow when conditions improve (large red cell) while other cells lyse to release their DNA (black chromosome), which may be taken up by different cells (blue and green cells). Science progresses in a similar way with some hypotheses being unpopular for years before returning to favour while parts of other, rejected, hypotheses may be incorporated into new ones.

One might therefore liken the dynamics of hypotheses to the dynamics of a population of bacteria that grow under favourable conditions and sporulate in an adverse environment or lyse to release DNA that may be taken up by others (Fig. 2). Alternatively, one might consider a hypothesis as existing on a chromatic scale of probabilities with its (varying) position determined by a (varying) consensus. Holding such views entails overcoming the obsession with avoiding being wrong at all costs, which underpins the “any jackass can trash a paper” comment (or project or grant application) (Drubin, 2011). In line with these views, one might also replace Wolfgang Pauli’s “not even wrong” with “not even interesting”. Indeed, one might argue that the function of prizes in general should be not so much about rewarding those whose findings have stood the test of time nor capturing public attention for some aspect of science nor even to support junior scientists; rather, they should be like the Turing Prize and encourage scientists to venture into the unknown and to think outside the box.

### Supplementary information

Peer Review File
