# Supplementary material for: Science and prizes: A case for rethinking the criteria for prizes in science (and for rewarding important discoveries in bacterial physiology)
Source: EMBO Rep. 2024 Feb 9;25(3):944–7. doi: 10.1038/s44319-024-00091-z (PMC10933288; doi:10.1038/s44319-024-00091-z)
Supplement: Supplementary file 1 — Peer Review File [file 44319_2024_91_MOESM1_ESM.pdf]

EMBO reports

Science and prizes

Vic Norris

Corresponding author(s): Vic Norris (victor.norris@univ-rouen.fr)

Review Timeline:

Submission Date:	31st Jan 24
Accepted:	1st Feb 24

Editor: Holger Breithaupt

Transaction Report: This manuscript was editorially reviewed.
